# Tuberculosis among Full-Time Teachers in Southeast China, 2005–2016

**DOI:** 10.3390/ijerph15092024

**Published:** 2018-09-17

**Authors:** Hongdan Bao, Kui Liu, Zikang Wu, Chengliang Chai, Tieniu He, Wei Wang, Fei Wang, Ying Peng, Xiaomeng Wang, Bin Chen, Jianmin Jiang

**Affiliations:** 1 School of Medicine, Ningbo University, Ningbo 315211, China; baohongdan@outlook.com (H.B.); wuzikang_cbg0@163.com (Z.W.); 2 Zhejiang Provincial Center for Disease Control and Prevention, Hangzhou 310051, China; Kliu@cdc.zj.cn (K.L.); chlchai@cdc.zj.cn (C.C.); hetieniu@sina.com (T.H.); jfwwang@cdc.zj.cn (W.W.); feiwang@cdc.zj.cn (F.W.); ypeng@cdc.zj.cn (Y.P.); xmwang@cdc.zj.cn (X.W.)

**Keywords:** full-time teachers, tuberculosis, China

## Abstract

*Objective*: To explore the incidence rate and characteristics of tuberculosis (TB) among full-time teachers from 2005 to 2016 in southeast China and to provide a basis for TB prevention and control measures in schools. *Methods*: Information about full-time teachers with TB was obtained from the National Tuberculosis Information Management System (NTIMS). Population data were collected from the Zhejiang Statistical Yearbook and the Zhejiang Education Yearbook. The TB incidence rates and epidemiological characteristics of full-time teachers were analyzed and the Chi-square test was used to analyze influencing factors of epidemiological characteristics and clinical characteristics, case-finding delay, and treatment outcomes. *Results*: A total of 1795 teachers with TB were reported from 2005 to 2016, and the annual incidence rate was 28.87 per 100,000. The average annual PTB (pulmonary TB) incidence rate among full-time teachers was 25.43/100,000 from 2005 to 2016 and the average annual PTB incidence rate among students was 15.40/100,000 from 2005 to 2016. The highest average incidence rates were observed in the QZ (Quzhou) and HZ (Hangzhou) districts. The male-to-female ratio of the patients was 0.95:1. Approximately half of the patients were 15–40 years old. The mean case-finding interval was 45.3 days. Multivariable logistic regression analysis of TB case-finding delay among full-time teachers revealed that the older (OR = 1.44, 95% CI = 1.18–1.76, *p* < 0.01), not local (OR = 1.81, 95% CI = 1.20–2.73, *p* < 0.01), retreatment (OR = 2.06, 95% CI = 1.39–3.08, *p* < 0.01) and extra-pulmonary tuberculosis (OR = 1.71, 95% CI = 1.13–2.61, *p* = 0.01) cases were at high risk of case-finding delay. Compared to physical examination, patients detected by referrals and tracking (OR = 2.26, 95% CI = 1.16–4.38, *p* = 0.02) and patients who directly visited the designated TB hospital (OR = 2.00, 95% CI = 1.03–3.88, *p* = 0.04) were more prone to case-finding delay. The cure rate of full-time teachers with TB was 77.10%. The cure rates differed significantly between groups classified based on age, case-finding patterns, diagnostic results, treatment classifications, and strategies of patient management. *Conclusion*: The TB incidence rate among full-time teachers decreased from 2005 to 2016, but teachers suffered a higher risk of TB than students. Western Zhejiang was a hotspot for TB incidence among full-time teachers. Female teacher and young and middle-aged teacher cases account for the majority of the reported patients. There was a case-finding delay among full-time teachers with TB. We should conduct regular physical examinations and strengthen full-course supervision to reduce the risk of TB patients with case-finding delay and increase the TB cure rate.

## 1. Introduction

Tuberculosis (TB) is the ninth leading cause of death worldwide and the leading cause of death from a single infectious agent, ranking above HIV/AIDS [1,2]. According to the 2017 Global Tuberculosis Report, 10.4 million people fell ill with TB in 2016, and 1.7 million people died from the disease (including 0.4 million people with HIV) [2]. China is one of 22 countries with the heaviest burdens of TB patients, with the third highest number of cases [2]. However, highly complex dynamics and spatial heterogeneity are associated with TB in China at the provincial level [3,4,5,6]. Zhejiang, as a province located in eastern China, is a highly socioeconomically developed province [7]. The province has 11 prefectures: HZ (Hangzhou), QZ (Quzhou), HUZ (Huzhou), JX (Jiaxing), SX (Shaoxing), ZS (Zhoushan), LS (Lishui), WZ (Wenzhou), TZ (Taizhou), JH (Jinhua), and NB (Ningbo). In recent decades, the TB incidence rate of Zhejiang province has decreased slowly from 2008 (71.50/100,000) to 2017 (47.54/100,000) and has remained at a modest level in China [7]. However, there are still 27,000 TB cases in Zhejiang every year due to the large population (55 million) [8].

According to a large number of school TB outbreak reports, clustering epidemics commonly occur, especially in senior schools and universities [9]. In China, students with TB accounted for 4.02% of total TB patients in 2014 [10], and TB outbreaks often occurred. Teachers who work in schools are at risk of being infected with TB or to becoming a source of infection [11]. For example, consider an outbreak that occurred in California. A teacher who developed multidrug-resistant TB (MDR-TB) was exposed to dozens of children, infecting 31 children with TB [12]. Therefore, it is important to monitor TB epidemics among full-time teachers. According to a study carried out in one prefecture of Zhejiang province, the average annual reported incidence rate of active TB among full-time teachers (39.15/100,000) was higher than that of active TB among students (22.34/100,000) from 2005 to 2011 [13]. Another study in one county of Zhejiang province also demonstrated that the average annual reported incidence rate of active TB among full-time teachers (25.42/100,000) was about 2.5 times higher than that of active TB among students (10.56/100,000) from 2005 to 2012 [14]. However, the TB incidence rate among full-time teachers in whole province has not been studied in China, and the baseline data is very limited. 

Based on the “National Tuberculosis Information Management System” (NTIMS) (established in 2005), we conducted this retrospective study among full-time teachers to (1) understand the baseline TB incidence rate among full-time teachers in Zhejiang province, (2) explore the epidemiological distribution and clinical characteristics of full-time teachers with TB and (3) provide evidence on TB prevention and control practices among full-time teachers in schools.

## 2. Data Collection

**Figure 1:**
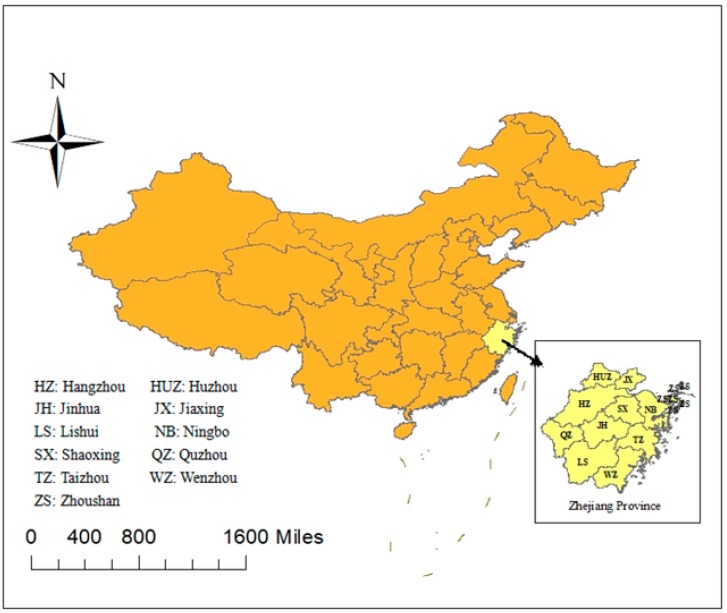
The location of Zhejiang province in China.

The NTIMS, which was established in 2005, provides a chance for us to analyze TB epidemics among full-time teachers in Zhejiang province (Figure 1) in the past 10 years. NTMIS records the demographic information, disease features, case-finding pattern and treatment outcome of each patient. We collected demographic and disease data for each teacher case with TB, including gender, age, nationality, habitation, sputum smear test results, time of TB symptom onset, time of diagnose, treatment classification, diagnose result, case-finding pattern, strategy of patient management and treatment outcome. The data we extracted covered the period from 1 January 2005 to 31 December 2016. We collected the annual population data for full-time teachers in 11 prefectures from the Education Yearbook of Zhejiang Province, which recorded the statistics for educational development, general administration of education, educational personnel management, basic and higher education and education examinations etc. [15]. Due to a lack of population data on the age and gender of full-time teachers in the Education Yearbook of Zhejiang Province, we are limited to conduct further analysis of TB incidence rates of different ages, genders or other demographic characteristics among full-time teachers. The annual population data for Zhejiang province were extracted from the Statistical Yearbook of Zhejiang Province, which recorded the population, finance, employment, education, and environment and resources, etc. [16].

### 2.1. Definitions

Case-finding delay: The time interval between the onset of TB symptoms and the first diagnosis of TB over 30 days [17].

Health service-seeking interval: The time interval between the onset of TB symptoms and the first to visit the health provider [18].

Diagnostic interval: The time interval between the first to visit the health provider and the first diagnosis of TB [18].

Case-finding pattern: In our study, the case-finding pattern mainly includes direct visits to the designated TB hospital, referrals and tracking and physical examinations.

Direct visit to a designated TB hospital: Patients directly visit the designated hospital for TB when they have symptoms.

Referrals and tracking: Referrals mean that patients who directly visited a non-designated hospital for TB were referred to a designated TB hospital; tracking refers to health workers tracking TB cases to designated TB hospitals who have not been referred to designated TB hospitals.

Physical examination: Here the physical examination means that teachers screened chest fluoroscopy for TB and recommended to designated TB hospitals if they are suspected as TB cases.

Treatment classification: Including initial treatment and retreatment. The initial treatment refers to patients treated for TB for the first time; retreatment refers to the patients who were cured from TB and diagnosed again for TB.

Habitation: Including local and not local. In our study, local means patients whose permanent residence registered in Zhejiang province; conversely, not local means patients whose permanent residence is registered out of Zhejiang province.

Onset time of symptoms: The time when patients felt the symptoms of TB.

Adverse outcomes included treatment failure, transfer to MDR-TB treatment, death, treatment interruption due to side effects, and loss to follow-up.

### 2.2. Statistical Analysis

The incidence rates of both TB and pulmonary TB (PTB) among full-time teachers, students and the total population were calculated based on the total population, the full-time teacher population and the population of students in schools. The PTB incidence rate of the three groups was also calculated in 11 prefectures and is shown in a disease map. Chi-square analysis was used to analyze the relationships between epidemiological characteristics and clinical characteristics, case-finding delay, and treatment outcomes.

Descriptive analysis and univariate analysis were performed by Statistical Package for Social Sciences (SPSS) version 18.0 (SPSS Inc., Chicago, Il, USA). The inspection level of the Chi-test is 0.05. Line graphs and columnar diagrams were generated by Microsoft Excel. The map of incidence rates was generated with ArcGIS (version 10.3, ESRI, Inc., Redlands, CA, USA).

### 2.3. Ethic Statement

Ethics approval of this study has been obtained from the Ethics Committee of Zhejiang Provincial Centers for Disease Control and Prevention (2014–2017). We declared that we have kept all the private information confidential.

## 3. Results

### 3.1. Basic Information about Full-Time Teachers with TB

There were 1795 full-time teachers with TB from 2005 to 2016 in Zhejiang province. With the 518,103 average annual number of full-time teacher, the average annual incidence rate was 28.87/100,000, and the PTB incidence rate was 25.43/100,000. Among those cases, there were 1559 PTB cases, including 438 (24.39%) sputum-smear-positive cases and 1121 (52.42%) sputum-smear-negative cases, 131 (7.29%) tuberculous pleurisy cases and 91 (5.07%) extra-pulmonary TB cases. Additionally, there were 14 (0.78%) cases without sputum test information.

### 3.2. Epidemiological Trend of the PTB Incidence Rate among Full-Time Teachers

**Figure 2:**
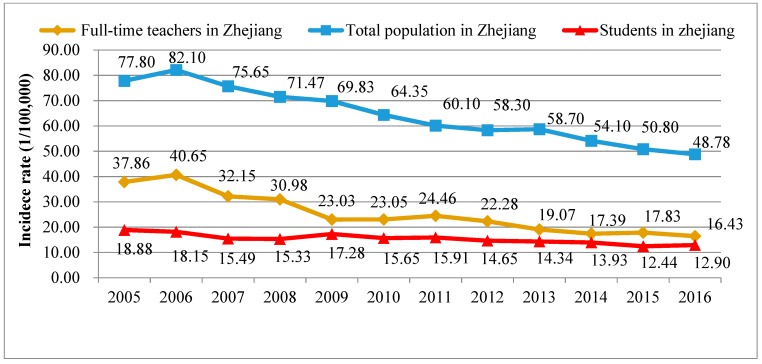
Pulmonary TB incidence rates among full-time teachers, students and the total population in Zhejiang.

The annual PTB incidence rate for the total population in Zhejiang province decreased gradually from 2005 (77.80/100,000) to 2016 (48.78/100,000), except for a small increase in 2013 (58.70/100,000). The annual PTB incidence rate among full-time teachers also decreased from 2005 (37.86/100,000) to 2016 (16.43/100,000), and this rate was lower than that among the total population in Zhejiang province. Small increases occurred in 2006 (40.65/100,000) and 2011 (24.46/100,000). These values were basically consistent with the trends of the total population. The annual PTB incidence among students decreased from 2005 (18.88/100,000) to 2016 (12.90/100,000) and was lower than that among full-time teachers (Figure 2).

### 3.3. Geographic Distribution of TB Cases among Full-Time Teachers

**Figure 3:**
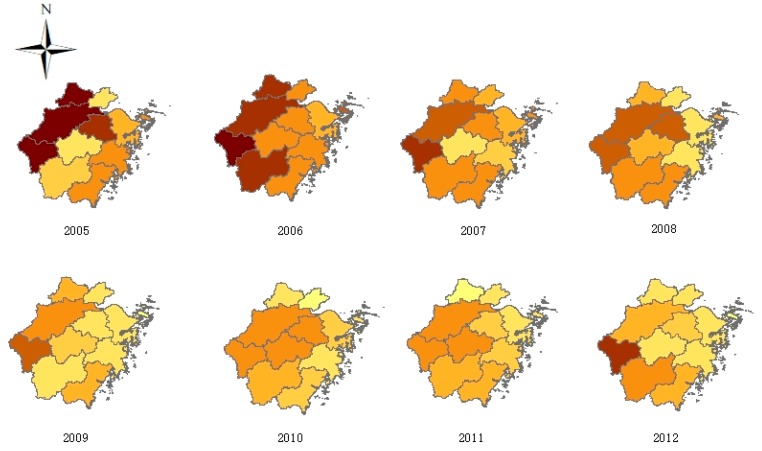

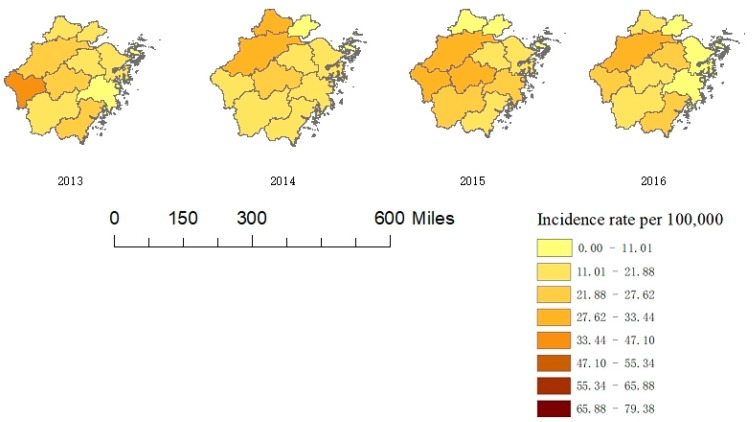
TB incidence rates among full-time teachers in Zhejiang province, 2005–2016.

The average annual TB incidence rates of different prefectures ranked from high to low were QZ (49.74/100,000), HZ (43.26/100,000), SX (31.37/100,000), LS (30.10/100,000), WZ (29.16/100,000), HUZ (27.19/100,000), JH (26.86/100,000), TZ (21.78/100,000), NB (20.39/100,000), ZS (18.79/100,000), and JX (16.99/100,000). The results showed that the incidence rates in the western part of the province were higher than those in the eastern part of the province; in addition, QZ had the highest incidence rate, and JX had the lowest incidence rate (Figure 3). 

### 3.4. Demographic Characteristics of TB Cases among Full-Time Teachers

Table S1 shows that over 50% of the full-time teachers with TB were less than 40 years old. Cases of TB among teachers greater than 60 years old accounted for the smallest percentage of cases.

Among the total cases, 874 patients were male, and 921 patients were female. The gender ratio (male to female) of all patients from 2005–2016 was 0.95:1. There were 1552 (86.46%) local teachers with TB, while 244 (13.59%) teachers with TB were not local. Han people contributed the most to all cases of TB among full-time teachers, accounting for 99.61% of such cases.

### 3.5. Associations between Clinical Characteristics of TB and Demographic Factors

Table S2 shows the relationships between the clinical classifications of TB and demographic factors. The proportion of smear-positive cases was lower among teachers between 30 and 60 years of age than among teachers less than 30 and greater than 60 years of age (*x*^2^ = 25.14, *p* < 0.01), and the proportion of retreatment cases was higher among male patients than among female patients (*x*^2^ = 11.20, *p* < 0.01). The retreatment case proportion was the highest among teachers over 60 years of age (*x*^2^ = 18.65, *p* < 0.01).

### 3.6. Case-Finding Pattern of Full-Time Teachers with TB

**Figure 4:**
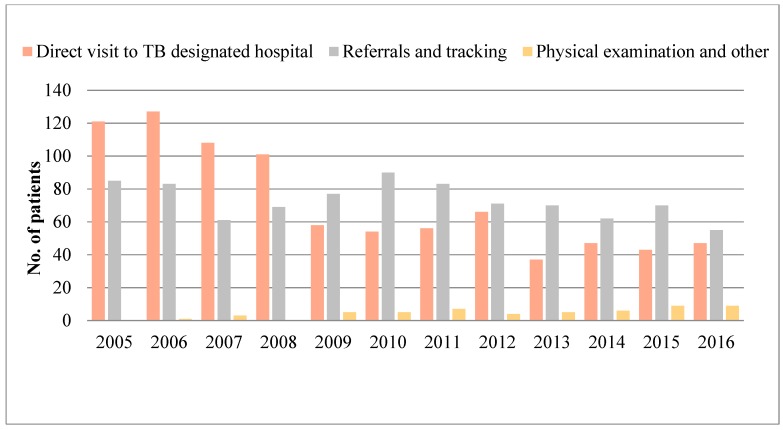
Case-finding patterns among full-time teachers in Zhejiang province, 2005–2016.

The reported number of TB cases detected by direct visits to designated TB hospitals decreased with some fluctuations from 2005 to 2016. The main TB case-finding pattern from 2005 to 2008 was direct visits to designated TB hospitals, but after 2009, the main methods of TB case finding were referrals and tracking. The TB cases detected by physical examination gradually increased after 2009 but still accounted for the smallest number of total TB cases (Figure 4).

### 3.7. Case-Finding Delay in TB Cases among Full-Time Teachers

Among all the 1795 cases, there are 1749 cases with the information of case-finding interval, and 46 cases have an information loss. Approximately 656 (37.51%) cases had a case-finding delay. The mean case-finding interval was 45.3 days, the mean health service-seeking interval was 37.4 days, and the mean diagnostic interval was 11.4 days. There were significant differences between the age, diagnostic result, case-finding pattern and treatment classification groups. The proportion of patients with case-finding delay increased with age (*x*^2^ = 24.46, *p* < 0.01), and the proportion of patients with case-finding delay was higher among retreated patients than among initially treated patients (*x*^2^ = 13.38, *p* < 0.01). Furthermore, the nonlocal population, patients who were detected by referrals or tracking and patients with extra-pulmonary TB had a higher proportion of case-finding delay (*x*_1_^2^ = 4.73, *p*_1_ = 0.03; *x*_2_^2^ = 6.16, *p*_2_ = 0.05; *x*_3_^2^ = 6.10, *p*_3_ = 0.05) (Table 1). 

We divided the group of age by median. To explore the factors influencing the case-finding delay, multivariable logistic regression analysis revealed that the older (OR = 1.44, 95% CI = 1.18–1.76, *p* < 0.01), not local (OR = 1.81, 95% CI = 1.20–2.73, *p* < 0.01), retreatment (OR = 2.06, 95% CI = 1.39–3.08, *p* < 0.01) cases were at high risk of case-finding delay. Compared to physical examination, patients detected referrals and tracking (OR = 2.26, 95% CI= 1.16–4.38, *p* = 0.02) and patients who directly visit the designated TB hospital (OR = 2.00, 95% CI = 1.03–3.88, *p* = 0.04) are more prone to case-finding delay. As for diagnostic results, extra-pulmonary TB cases have a higher risk than pulmonary TB cases (OR = 1.71, 95% CI = 1.13–2.61, *p* = 0.01) (Table 2).

### 3.8. Treatment Outcomes of Full-Time Teachers with TB

Among all the TB cases in full-time teachers, the cure rate of full-time teachers with TB was 77.10%. Table 3 shows the relationships between treatment outcome and its influencing factors. There were significant differences between the groups based on treatment classifications, diagnostic results, ages, case-finding patterns and strategies of patient management. The cure rate decreased with age (*x*^2^ = 20.71, *p* < 0.01). Patients with PTB, patients detected by clinical consultation and patients undergoing initial treatment had relatively high cure rates (*x*_1_^2^ = 82.83, *p*_1_ < 0.01; *x*_2_^2^ = 71.42, *p*_2_ < 0.01; *x*_3_^2^ = 9.41, *p*_3_ < 0.01). Furthermore, patients with self-administration had a far lower cure rate than patients with full-course supervision (*x*^2^ = 927.62, *p* < 0.01) (Table 3).

## 4. Discussion

By applying data from the NTIMS, our study is the first to present the epidemiological and clinical characteristics of TB among full-time teachers in China. We found that the incidence rate of TB among full-time teachers decreased during recent decades in Zhejiang province. According to the World TB Report 2017, the number of TB cases and the incidence rate of TB decreased gradually in China [2]. The TB situation in Zhejiang province, among both the total population and full-time teachers, is consistent with this trend [8], which indicates that TB prevention and control have achieved some progress in recent years [8,19]. The average PTB incidence rate was approximately 2.67 times lower than that of the total population. Compared to teachers, there are some other occupational groups such as farmers and migration workers with poor socioeconomic status having a higher TB incidence rate. On the other hand, there are few people over 65 years old among teachers and students while age over 65 was a risk factors of the TB incidence [20]. Although teachers had a lower risk of TB than the total population, the average PTB incidence rate among full-time teachers from 2005 to 2016 was approximately 1.65 times higher than that of students, which indicated that teachers were at higher risk of TB than students in schools.

The results showed the geographical distribution of TB among full-time teachers in 11 regions of Zhejiang. The distribution pattern (high in the west and low in the east) was basically consistent with the distribution of TB among the total population in the province and the country [7,21]. Western Zhejiang, which mainly includes QZ and HZ, was a hotspot for TB among teachers. Accordingly, QZ has one of the highest disease burdens of TB in Zhejiang province [21]. This high incidence may be related to the large proportion of agricultural populations and the relatively low economic development in QZ compared with those in other parts of Zhejiang [22]. Hangzhou, as the capital city of Zhejiang, which has more migration workers from the western part of China, has a high incidence rate of TB among full-time teachers. In contrast, eastern Zhejiang, such as ZS, NB, and JX, which are located in the eastern coastal areas of Zhejiang province, have a high level of economic development [22] and a low incidence rate of TB among both the total population and full-time teachers. 

The results showed that the M:F ratio among full-time teachers with TB was 0.95:1, which is consistent with the results of Jun Li et al. [13]. This ratio is very different from the M:F ratio among the total population [20,23,24]. These results could be explained by the fact that female teachers account for the majority of the teacher population. Regarding age distribution, our study found that young and middle-aged teachers accounted for the majority of cases. The distribution is similar to that of TB among the total population [25].

In general, direct visits to a designated TB hospital were the major case-finding mode for teachers with TB, followed by referrals or tracking. Only 3.00% of cases were detected by physical examinations. In fact, chest fluoroscopy for TB is a mandatory part of the physical examination for teachers in China [26]. According to relevant rules, teachers in China will be suspended from work if they have TB [26]. Therefore, teachers who are suspected to have TB may conceal their results and prolong the visiting period. Because early detection of TB is emphasized in the World Health Organization (WHO) End-TB strategy and teachers with TB could be an infection source for students in schools [27,28], we suggest that both schools and Centers for Disease Control (CDC) should take more measures to monitor and report full-time teachers with TB.

The case-finding interval was influenced by many factors, which mainly involved patients and medical institutions [29,30]. Our results show that 35% of full-time teachers with TB had a case-finding delay, and the mean case-finding interval was 45.3 days. The mean health service-seeking interval was 37.4 days, and the mean diagnostic interval was 11.4 days. Patients who are not diagnosed in a timely manner will be contagious for a long period of time, which will greatly increase the risk of group transmission [31]. According to previous studies, the case-finding delay among full-time teachers with TB is shorter than that among typical PTB patients [25,32,33], and the mean diagnosis interval was also shorter than those of PTB patients in Taiwan [34], tuberculous meningitis patients [35], and active TB patients after kidney transplantation [36]. The case-finding interval among full-time teachers with TB was longer than that among PTB patients in Zhejiang province [37]. In fact, both the case-finding interval and the health service-seeking interval among full-time teachers with TB were over 1 month, which accounts for most of the total delay. This phenomenon indicates that awareness about seeking health care needs to be improved among full-time teachers with TB. Moreover, our study showed that the proportion of case-finding delays increased with age. On the one hand, older teachers are more susceptible to chronic bronchitis, pneumonia and other diseases [38]. Because the symptoms of PTB are difficult to distinguish from these diseases, TB is difficult to diagnose. On the other hand, poor knowledge about TB leads to delayed care-seeking [18]. Young people may receive more TB health education in the new era of the internet. Such patients have more access to health services by using new technologies, such as mobile phone appointment systems. Our study also found that retreatment cases have a significantly higher proportion of case-finding delays than initial treatment cases. The initial treatment failure or side effects of drugs could lead retreated patients to lose confidence in treatment and cause a delay in seeking health care [39,40]. Regarding case-finding patterns, we found that patients detected by referrals or tracking and clinical consultations were prone to being delayed. Compared with physical examinations, referrals involve a more complicated process, which involves information transfer, personnel coordination and other factors, increasing the risk of a case-finding delay. The results of our study suggest that physical examinations could be an effective way to reduce this delay. It is also necessary to provide more health education and psychological counseling for retreatment patients to decrease delays. 

The cure rate of full-time teachers with TB in our study was 77.10%, which is lower than that reported among all TB cases in China [41] and in other countries [42,43]. The results showed that the cure rate of patients under full-course supervision is significantly higher than that of patients under self-administration. As members of an aggregated group, teachers need better treatment to recover early. Due to poor self-administration, it is necessary to strengthen the supervision and treatment of teachers with TB. Moreover, patients with different TB disease categories also differed in terms of cure rate. The cure rate of extra-pulmonary TB cases was lower than that of PTB and tuberculous pleurisy cases. Similar to PTB patients among the total population, retreatment TB cases among full-time teachers had a lower cure rate than initial treatment TB cases [44]. Retreatment cases are at high risk of diagnostic delay [45], and approximately 24% of cases will be transferred to MDR-TB [46], thus contributing to the decreasing cure rate. With increasing age, the cure rate of full-time teachers with TB decreased. Aged patients are more prone to poor medication adherence and treatment adherence [47]. In addition, some aged patients cannot seek health care in a timely manner due to limited mobility. These results indicate that we should pay more attention to the treatment and follow-up of older teachers with TB and retreatment patients with TB.

## 5. Limitations

First, our study did not conduct a deep analysis of the incidence rate of TB among teachers of different ages, genders or other demographic characteristics due to a lack of population data on the age and gender of full-time teachers. Second, due to the sensitivity of TB disease for teachers, some teachers with TB may not be reported, leading to an underestimation of this epidemic. Finally, due to the lack of geographic location of case, we conducted the multiple logistic regression without residual spatial correlation analysis, which may lead to a bias of the model.

## 6. Conclusions

In conclusion, the incidence rate of TB among teachers gradually decreased from 2005 to 2016.Teachers suffered a higher risk of TB than students and a lower risk of TB than the total population. Since the teachers are more likely to be the infection source of school TB outbreaks, TB epidemic among full-time teachers is worthy of monitoring. Western Zhejiang was a hotspot for TB incidence, and it is necessary to strengthen physical examinations for TB in those regions to monitor the TB epidemic among teachers. Female teacher and young and middle-aged teacher cases account for the majority of the reported patients. There was a case-finding delay among full-time teachers with TB. Conducting regular physical examinations and strengthening full-course supervision could reduce the risk of case-finding delays and increase the TB cure rate. Intensive patient care and follow-up service should be provided for elderly teachers with TB.

## Figures and Tables

**Table 1:** Univariate analysis of case-finding delays among full-time teachers with TB.

Index	Case-Finding Delay		*x* ^2^	*p*
	Yes	No		
Gender				
Male	324 (37.90)	531 (62.10)	0.12	0.74
Female	332 (37.10)	562 (62.90)		
Age				
<30	177 (30.70)	399 (69.30)	25.65	<0.01 **
30–39	179 (37.60)	297 (62.40)		
40–49	107 (37.90)	175 (62.10)		
50–59	120 (46.50)	138 (53.50)		
≥60	73 (46.50)	84 (53.50)		
Habitation				
Local	607 (36.90)	1039 (63.10)	4.73	0.03 *
Not local	49 (47.60)	54 (52.40)		
Onset time of symptoms				
First quarter	176 (42.40)	239 (57.60)	5.94	0.11
Second quarter	175 (37.00)	298 (63.00)		
Third quarter	152 (35.80)	273 (64.20)		
Fourth quarter	153 (35.10)	283 (64.90)		
Treatment classification				
Initial treatment	598 (36.40)	1045 (63.60)	14.26	<0.01 **
Retreatment	58 (54.70)	48 (45.30)		
Case-finding patterns				
Direct visit to designated TB hospital	308 (37.10)	523 (62.90)	6.16	0.05 *
Referrals or tracking	336 (38.90)	528 (61.10)		
Physical examination	12 (22.20)	42 (77.80)		
Diagnostic result				
Pulmonary tuberculosis	558 (36.70)	963 (63.30)	6.10	0.05 *
Tuberculous pleurisy	49 (38.30)	79 (61.70)		
Extra-pulmonary tuberculosis	49 (49.00)	51 (51.00)		

Note: *: *p* < 0.05, **: *p* < 0.01.

**Table 2:** Multivariable logistic regression analysis of case-finding delays among full-time teachers with TB.

Index	*B*	OR (95% CI)	*p*
Age			
≤35	--	1	--
>35	0.37	1.44 (1.18, 1.76)	<0.01 **
Habitation			
Local	--	1	--
Not local	0.60	1.81 (1.20, 2.73)	<0.01 *
Treatment classification			
Initial treatment	--	1	--
Retreatment	0.73	2.06 (1.39, 3.08)	<0.01 **
Case-finding patterns			
Physical examination	--	1	--
Direct visit to designated TB hospital	0.69	2.00 (1.03, 3.88)	0.04 *
Referrals or tracking	0.81	2.26 (1.16, 4.38)	0.02 *
Diagnostic result			
Pulmonary tuberculosis	--	1	--
Tuberculous pleurisy	0.05	1.05 (0.72, 1.53)	0.79
Extra-pulmonary tuberculosis	0.54	1.71 (1.13, 2.61)	0.01 **

Note: *: *p* < 0.05, **: *p* < 0.01.

**Table 3:** Univariate analysis of treatment outcomes among full-time teachers with TB.

Index	Treatment Outcome		*x* ^2^	*p*
	Cure	Adverse Outcomes		
Age				
<30	481 (81.94)	106 (10.06)	20.71	<0.01 **
30–39	384 (78.21)	107 (21.79)		
40–49	212 (74.39)	73 (25.61)		
50–59	195 (73.58)	70 (26.42)		
≥60	112 (67.07)	55 (32.93)		
Gender				
Male	666 (76.20)	208 (23.80)	0.78	0.38
Female	718 (78.00)	203 (22.00)		
Habitation				
Local	1299 (76.80)	393 (23.20)	1.82	0.18
Not local	85 (82.50)	18 (17.50)		
Case-finding patterns				
Clinical consultation	592 (68.40)	273 (31.60)	71.42	<0.01 **
Referrals and tracking	748 (85.40)	128 (14.60)		
Physical examination	44 (81.50)	10 (18.50)		
Case-finding delay				
Yes	517 (78.80)	139 (21.20)	0.01	0.94
No	863 (79.00)	230 (21.00)		
Result of sputum				
Smear positive	357 (81.50)	81 (18.50)	0.95	0.33
Smear negative	889 (79.30)	232 (20.70)		
Diagnostic result				
Pulmonary tuberculosis	1246 (79.92)	313 (20.08)	82.83	<0.01 **
Tuberculous pleurisy	94 (71.76)	37 (28.24)		
Extra-pulmonary tuberculosis and others	44 (41.90)	61 (58.10)		
Treatment classification				
Initial treatment	1313 (77.88)	373 (22.12)	9.41	<0.01 **
Retreatment	71 (65.14)	38 (34.86)		
Strategy of patient management				
Full-course supervision	1338 (91.50)	124 (8.50)	927.62	<0.01 **
Self-administration or other	46 (13.80)	287 (86.20)		

Note: **: *p* < 0.01.

## Supplementary Materials

The following are available online at http://www.mdpi.com/1660-4601/15/9/2024/s1, Table S1: Age composition of reported tuberculosis cases among full-time teachers in Zhejiang province, 2005–2016, Table S2: Relationships between clinical classifications of TB and demographic factors.
