# Supplementary material for: Tuberculosis among Full-Time Teachers in Southeast China, 2005–2016
Source: Int J Environ Res Public Health. 2018 Sep 17;15(9):2024. doi: 10.3390/ijerph15092024 (PMC6163467; doi:10.3390/ijerph15092024)
Supplement: Supplementary file 1 [file ijerph-15-02024-s001.pdf]

Table S1. Age composition of reported tuberculosis cases among full-time teachers in Zhejiang province, 2005-2016.

Year	<30		30–39		40–49		50–59		≥60	
	No. of Patients	Proportion (%)	No. of Patients	Proportion (%)	No. of Patients	Proportion (%)	No. of Patients	Proportion (%)	No. of Patients	Proportion (%)
2005	66	32.04	50	24.27	33	16.02	26	12.62	31	15.05
2006	68	32.23	59	27.96	24	11.37	34	16.11	26	12.32
2007	54	31.40	32	18.60	25	14.53	38	22.09	23	13.37
2008	51	30.00	41	24.12	25	14.71	31	18.24	22	12.94
2009	41	29.29	37	26.43	28	20.00	20	14.29	14	10.00
2010	45	30.20	48	32.21	25	16.78	18	12.08	13	8.72
2011	63	43.15	28	19.18	26	17.81	22	15.07	7	4.79
2012	51	36.17	45	31.91	25	17.73	13	9.22	7	4.96
2013	43	38.39	35	31.25	17	15.18	12	10.71	5	4.46
2014	37	32.17	34	29.57	17	14.78	19	16.52	8	6.96
2015	37	30.33	46	37.70	16	13.11	18	14.75	5	4.10
2016	31	27.93	36	32.43	24	21.62	14	12.61	6	5.41
Total	587	32.68	491	27.34	285	15.87	265	14.76	167	9.30

Table S2. Relationships between clinical classifications of TB and demographic factors.

**Factors**	**Clinical Classification of TB**							
	**Smear Positive (%)**	**Smear Negative (%)**	x^2	***P***	**Initial Treatment**	**Retreatment**	x^2	***P***
Gender								
Male	226 (29.70)	535 (70.30)	1.89	0.17	804 (92.00)	70 (8.00)	11.20	<0.01**
Female	212 (26.60)	586 (73.40)			882 (95.80)	39 (4.20)		
Age								
<30	151 (28.80)	373 (71.20)	25.14	<0.01	563 (95.90)	24 (4.10)	18.65	<0.01**
30-39	96 (22.50)	330 (77.50)			467 (95.10)	24 (4.90)		
40-49	65 (27.50)	171 (72.50)			267 (93.70)	18 (6.30)		
50-59	61 (27.10)	164 (72.90)			242 (91.30)	23 (8.70)		
≥60	65 (43.90)	83 (56.10)			147 (88.00)	20 (12.00)		
Habitation								
Local	410 (27.90)	1057 (72.10)	0.26	0.61	1588 (93.90)	104 (6.10)	0.28	0.59
Not local	28 (30.40)	64 (69.60)			98 (95.10)	5 (4.90)		

Note: ** $p < 0.01$.
